# Plant Cell-on-Chip (PCOC): Exploring the Electrical Modulation Capability of Plant Cells

**DOI:** 10.3390/mi17091015

**Published:** 2026-08-27

**Authors:** Jiayu Li, Ruyu Zhou, Yuxiang Qin, Xiuyun Liu, Kewei Liu, Miao Yu, Xiang Ren

**Affiliations:** 1 Department of Microelectronics, Tianjin University, Tianjin 300072, China; 2 Department of Applied and Computational Mathematics and Statistics, University of Notre Dame, Notre Dame, IN 46556, USA; rzhou2@nd.edu; 3 Department of Computer Science & Statistics, University of Rhode Island, Kingston, RI 02881, USA; 4 Tianjin Key Laboratory of Imaging and Sensing Microelectronic Technology, Tianjin University, Tianjin 300072, China; 5 State Key Laboratory of Advanced Materials for Intelligent Sensing, Tianjin University, Tianjin 300072, China; 6 Medical School, Tianjin University, Tianjin 300072, China; 7 State Key Laboratory of Advanced Medical Materials and Devices, Tianjin 300072, China; 8 School of Pharmaceutical Science and Technology, Tianjin University, Tianjin 300072, China; 9 The Sino-German College of Intelligent Manufacturing, Shenzhen Technology University, Shenzhen 518118, China; 10 Department of Research and Development, Stedical Scientific, Carlsbad, CA 92010, USA; 11 Key Laboratory of Organic Integrated Circuit, Ministry of Education, Tianjin University, Tianjin 300072, China

**Keywords:** Plant Cell-on-Chip (PCOC), electrochemical impedance spectroscopy, synthetic biology, microfluidics

## Abstract

The intrinsic properties of plants offer numerous opportunities for scientific and technological advancement. Considerable efforts have been directed toward developing plant-on-chip platforms to investigate cellular responses to external stimuli, including chemical, mechanical, and electrical cues. In this study, we present a fluidic platform using polydimethylsiloxane (PDMS) and a printed circuit board (PCB), integrated with electrochemical impedance spectroscopy (EIS) detection. Various experimental conditions were examined, including ionic and pH stimulation, as well as membrane dimensions, with the onion inner membrane treated as a black-box system. The measurement results are presented as Nyquist plots, and a resistance model incorporating multifactorial influences is proposed. Impedance variations in plant cells serve as a basis for electrical modulation. To explore these properties, we converted acoustic signals into electrical inputs and recorded the outputs after being modulated by onion inner epidermal cells. A transfer function analysis was subsequently performed. Our results indicate that the plant cell-on-chip (PCOC) platform holds promise for further investigations into plant cell properties. The impedance results suggest that plant cells can respond to different external stimuli, enabling modulation of the electrical properties. These findings lay the groundwork for future studies on cellular electrical characteristics and the development of preliminary bioelectrical circuits.

## 1. Introduction

Plant cells respond to their environment in unique ways, particularly through their membranes, which are directly exposed to external conditions. Changes in ionic composition, pH, and electrical stimulation can alter cellular activity, resulting in dynamic variations in impedance.

Current studies on the electrical properties of plant cells are separated into mechanical and electrical analyses. Mechanical approaches focus on cellular deformation and the gating mechanisms of mechanosensitive ion channels, attempting to explain the sensing ability of plants from mechanical force and deformation to ion flux [1,2], while electrical approaches employ electrochemical impedance spectroscopy (EIS) to obtain tissue impedance spectra and establish equivalent circuit models, simplifying cells as resistors and capacitors for assessing membrane integrity and physiological states [3].

Between these two directions, a systematic bridge is notably absent. The few attempts to integrate mechanical and electrical analyses have largely been confined to the piezoelectric effect, which mainly focuses on the direct generation of charge separation by mechanical stress [4]. Researchers point out that the piezoelectric effect in living plant tissues is unlikely to produce sustained electrical signals [4]. This result suggests that the current analytical framework is insufficient to explain the rich electrical behaviors exhibited by plant cells under complex physiological conditions.

The impedance response of plant cells is inherently a result of multiphysics coupling. Although equivalent circuit models can fit the observed impedance spectra through parameter-fitting methods, they struggle to explain the physiological and physical meanings of these electrical components. Current analytical approaches tend to simplify the impedance response of plant cells, abstracting cellular states into a few electrical parameters. While this is efficient for engineering diagnostics, it sacrifices substantial information about the underlying physical mechanisms. This limitation constitutes the core bottleneck in applying plant cell electrical characterization research to bioelectronic applications.

Another limitation is that current electrophysiological studies are largely confined to a single direction, detecting cellular states from electrical information. The reverse research perspective remains rarely explored, which involves controlling cell states to achieve targeted modulation of electrical properties.

Targeted modulation of plants’ electrical properties enables plant cells to serve as foundational components, which can be a proof of concept for preliminary synthetic circuitry in the semiconductor synthetic biology (SemiSynBio) field. SemiSynBio is an emerging field that integrates synthetic biology with semiconductor technology to harness biological systems for computational purposes. It leverages the inherent properties of biomaterials to develop self-organizing, resilient, and adaptive computing networks. By utilizing cellular or synthetic cell-based systems for information processing and storage, SemiSynBio aims to overcome the limitations of conventional semiconductor methods. Plant cells are particularly well suited for exploring along the SemiSynBio path due to their ease of handling, simple structure, and robust vitality. Their ability to respond to external stimuli by changing impedance or modulating signal responses makes them valuable models for advancing this field. Integrating cells into circuits as functional components enables the construction of hybrid computing networks that are both biological and electronic. The use of biomaterials as electronic components, referred to as living electronics, offers distinct prospects and advantages.

The current state of hybrid systems is still in its very basic and preliminary stage. Biological materials have been used to achieve functions such as wires, capacitors, transistors, and diodes, among others [5,6,7]. A typical application is the plant wire. For example, lettuce seedlings have been connected to circuits to obtain impedance and direct current transfer function measurements [8]. The plant-wire research shifts the focus of PCOC from studying plant characteristics to exploring the potential of plants for applications in circuits, bringing it closer to the goals of semiconductor synthetic biology. But the function that plant cells achieve is still confined to transmitting electrical signals, neglecting how electrical signals will be modulated through these cells and whether the modulation ability of plant cells can be designed. The current understanding of plant cell mechanobiology and electrical signaling in SemiSynBio field is limited and not developed enough to support SemiSynBio integration.

The complex microstructure of living cells endows them with distinct impedance features, including resistive and capacitive properties. When current flows through, the influence of cellular structure and ion exchange enables living cells to function as complex integrated living electronics, analogous to an integrated semiconductor chip with specific functions.

Plant cells possess a more complex impedance structure than isolated biological membranes while also exhibiting clearer structural and mechanistic features than animal cells. Take the inner epidermal cells of onions as an example. These cells contain structures such as cell membranes, cell walls, and vacuoles, each contributing to the overall resistance and capacitance of the system, influencing its modulation effects. These effects can be controlled through the cellular responses to external stimuli. These properties provide the opportunity to explore the use of plant cells as electrical components in synthetic circuitry. In the development of such electrical components, exploring the electrical characteristics of plant cells and verifying their possibilities is a necessary first step that guides our experiment and analysis.

In the research on plant cell properties, the PCOC platform has greatly facilitated experimental implementation and research advancement and has become our method for verifying the synthetic circuitry concept. The convergence of microfluidics and chip technology has led to the development of PCOC, a label-free and versatile platform for investigating plant cells [9]. PCOC integrates plant cells with electrical circuits, enabling either the construction of circuits with specific functions using plant components or the investigation of plant properties by incorporating plants into the circuit. By combining plant cells with circuits and various instruments, PCOC allows for the measurement and analysis of cellular electrical performance under diverse conditions, offering a flexible tool for plant research. This platform can effectively reduce the influence of the plant growth environment and enables more accurate measurements at both the tissue and cellular levels. For example, electrical stimulation applied through electrodes can clearly reveal the electrical responses of sensitive plants such as Mimosa [10].

Lab-on-chip platforms combine the miniaturization advantages of chips with the flexibility and controllability of fluid manipulation. In synthetic biology, lab-on-chip devices are extensively used for culturing and investigating animal cells [11], developing organotypic microfluidic models for glioma [12], and enabling digital microfluidic platforms for diagnosis [13], demonstrating broad potential and adaptability. These lab-on-chip methodologies have also been successfully extended to plant cell research. By integrating microfluidics, microscale fluid control allows real-time observation of plant cell growth under various environmental conditions and facilitates measurement of key parameters such as mechanical properties and pollutant tolerance [14]. Building on these capabilities, PCOC approaches have been introduced to investigate diverse plant systems, including roots [15], tip-growing cells [16,17], algae [18], and other plant cell types [19]. Collectively, these examples highlight the ability of PCOC to provide controlled microenvironments, enable quantitative analysis, and support real-time monitoring, features that distinguish it from conventional plant cell assays.

Electrochemical impedance spectroscopy (EIS) enables precise monitoring of plant cell processes because the electrical properties of plant cells change in response to external stimuli and cellular activities. EIS workstations integrated into PCOC devices have demonstrated advanced capabilities for real-time monitoring [20]. In addition, innovative combinations of microfluidic devices with printed circuit boards (PCBs) have simplified the measurement of membrane electrical properties [21], an approach that can also be applied to plant cells.

Despite these advances, the use of EIS-integrated PCOC in plant cell research remains underexplored, and changes in plant cell electrical behaviors under varying environmental conditions have yet to be quantified. Further research in this area could yield significant breakthroughs and insights, positioning EIS-integrated PCOC as a powerful and easy-to-use tool for plant science.

Most existing research on plant characteristics using EIS focuses on the whole plant, with few studies targeting specific plant structures. Most studies on plant properties using EIS focus on growth performance, chemical distribution, and biological and chemical characteristics, which are important in the field of plant science [22]. Our emphasis lies in broadening the application of biological materials in electrical engineering, so we concentrate on a tissue composed of a single cell type. Analyzing plant membranes offers distinct advantages for electrical applications, including miniaturization, enhanced operability, structural simplification, and greater convenience for subsequent device integration. Moreover, tissue-level analysis reduces the analytical difficulties arising from the complexity of plant organs, allowing us to preserve the intrinsic advantages of plant cells, such as self-repair and active responsiveness, and to investigate their fundamental properties from a more elementary starting point. Exploring the electrochemical behavior of distinct plant structures is not only helpful for understanding plant composition but also for elucidating internal reaction mechanisms.

The integration of PCB chips with EIS detection can improve the accuracy of membrane tissue measurements to a certain extent. PCOC provided us with the inspiration to develop devices and methods to explore the electrical properties of plant cells for potential new applications. We designed a fluidic platform integrating a PDMS and PCB-based chip with EIS detection to study the response of onion inner epidermal cell membranes when stimulated by different ionic liquids and to analyze the electrical modulation ability of the onion inner layer, providing a preliminary verification of the SemiSynBio concept.

## 2. Materials and Methods

### 2.1. LOC Device Design

**Figure 1:**
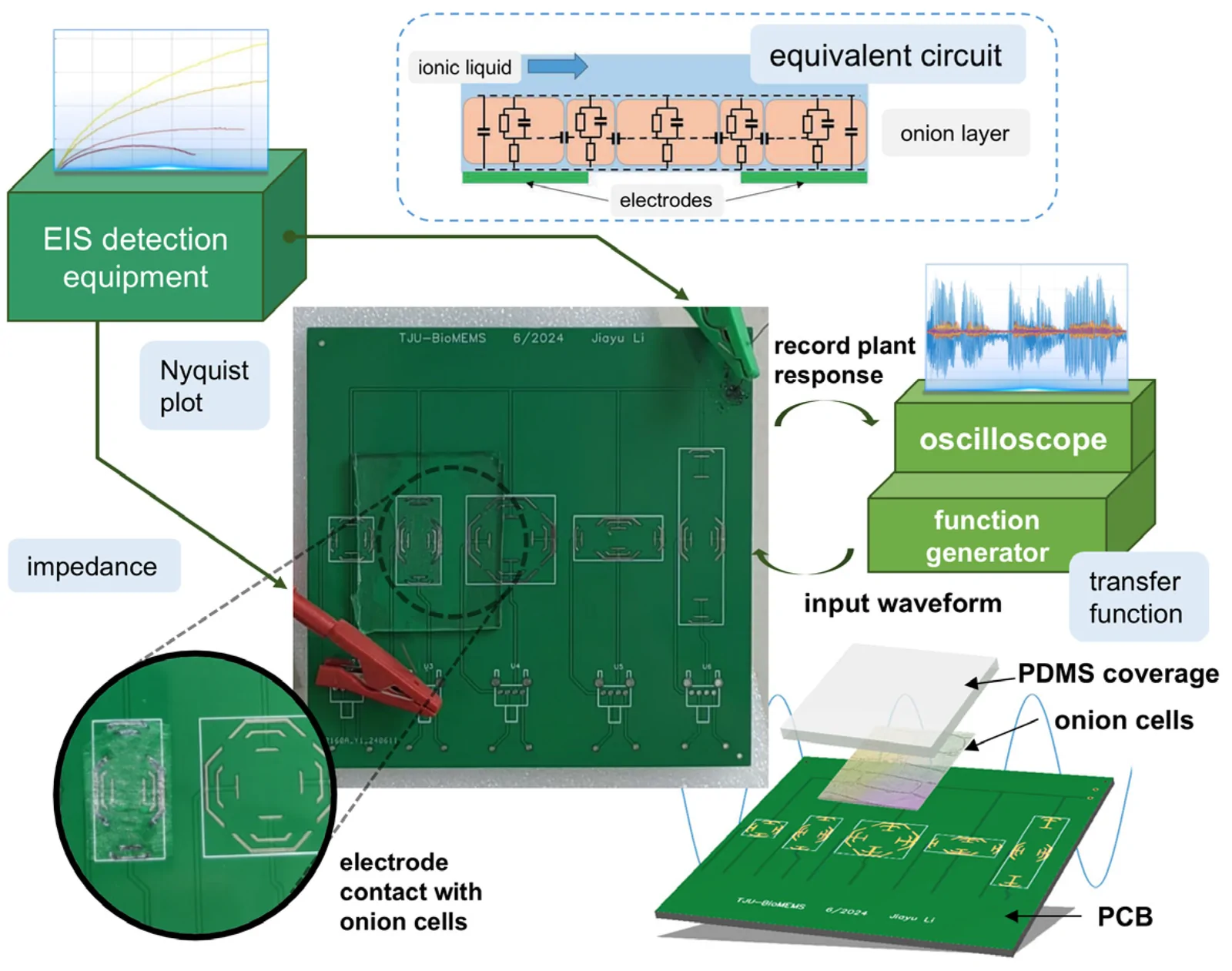
The PDMS- and PCB-based device.

To perform EIS tests on onion inner epidermal membranes of varying dimensions under different conditions, we designed a reconfigurable PDMS- and PCB-based device consisting of two parts. The PDMS- and PCB-based device is shown in Figure 1. The bottom PCB serves as the foundational support and provides electrical signal input. It was manufactured by Shenzhen JLC Technology Group in China. The overall area of the PCB is 100 × 100 mm^2^. Five contact areas are designed on the PCB with different sizes, including 10 × 10, 10 × 20, 20 × 10, 20 × 20, and 40 × 10 mm^2^. The geometric structure of the central pad was designed to establish suitable contact between the cells and the circuit system. We adopted two or three polygons on each side, and the length and width of the contact electrodes were adjusted from 10 mm to 20 mm according to the measurement areas. The design of the electrode was based on a variant of the ring-shaped interdigital electrode. By ensuring that the membrane has a certain transmission path, this structure increases the total edge length of the electrode, generating a sufficiently strong local electric field. It can ensure that the current path flows through the cells. The PDMS piece on top is used for fixing and sealing. We utilized flat PDMS without microstructure. To ensure a stable electrical seal and prevent solution leakage between the electrodes, the PDMS layer was mechanically clamped with constant pressure. Data were acquired using the EIS instrument (Admiral Instruments Squidstat Plus Potentiostat, Tempe, AZ, USA). The frequency range of this platform is determined by the EIS instrument from 10 Hz to 1 MHz.

### 2.2. Onion Membrane Operation

The onion inner membrane serves as an ideal system for impedance studies due to its operability and cell viability. Using a microsurgical approach, we cut onion blocks to appropriate dimensions and directly stripped the inner epidermal cells. The cells were maintained in 1× PBS to preserve viability. We observed the stripped onion layer under the microscope to ensure the inner membrane was composed of a single cell layer.

To investigate dimension-dependent resistance, we selected onion membranes of various sizes, including 10 × 10, 10 × 20, 20 × 10, 20 × 20, and 40 × 10 mm^2^. Each membrane was positioned flat on the chip to establish direct contact between the cells and the electrode pad and covered with the flat PDMS. Before placing the membrane on the electrodes, we used lint-free paper to ensure that there was no residual ionic solution on the side in contact with the electrodes. We used a mechanical fixture to clamp the PCB, onion layer and PDMS, ensuring no movement and tight contact. Each experiment was conducted following these steps, including the complex signal analysis experiment.

Treating the onion inner membrane as a black-box system, we applied sinusoidal signals across a range of frequencies (potentiostatic electrochemical impedance spectroscopy, PEIS) to perform impedance measurements. The resulting impedance changes were visualized using Nyquist plots.

Onion epidermal cells respond differently to changes in the concentration of ions such as potassium, sodium, and calcium, for instance, by altering ion pump activity. To study these responses, we treated the onion epidermal cells with KCl solutions of varying concentrations and with liquids of different pH levels.

Following EIS measurement, plasmolysis and recovery tests confirmed that the plant cells remained viable under suitable KCl concentrations. All experiments were performed within strict time limitations to ensure consistency. We set 2 min for onion layer operation and 1 min for ion liquid treatment, with testing time limited to 10 min. During experimental measurements, we performed three consecutive measurements for each condition. The differences among the three measurements for each curve were negligible; therefore, each curve shown in the figures represents the average of three consecutive tests.

## 3. Result

### 3.1. Dimensions

**Figure 2:**
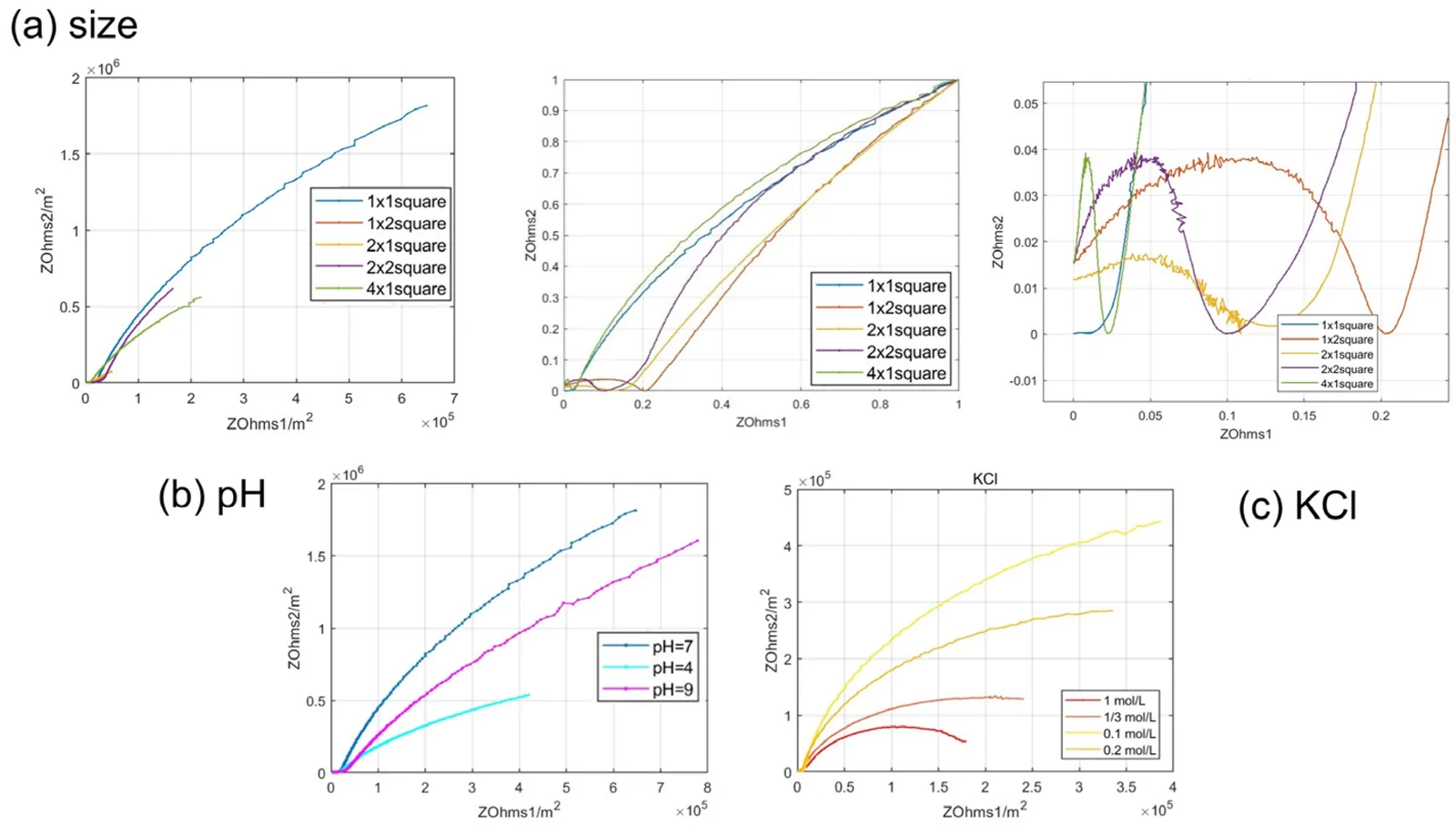
Re vs. -Im Nyquist plot of various dimensions, pH stimulations and KCl stimulations. Reproduced with permission [23]. Copyright © 2025, IEEE.

Figure 2 presents the results obtained from the electrochemical impedance measurements. For different cell areas, we defined the input side as W (width) and the other side as L (length) and displayed the data using complex Nyquist plots (real vs. negative imaginary components). The W and L dimensions of the membrane influence the distribution and propagation of the electrical stimulus, thereby affecting the resistance. These resistance changes were detected via electrochemical impedance measurements. As shown in Figure 2a, the impedance curves varied significantly across membranes with different areas and aspect ratios. We normalized the five datasets to a range of 0 to 1 for presentation. At high frequencies (typically above 5 kHz), the curves exhibited similar geometric features but with distinct peaks.

### 3.2. pH

Plant cells typically maintain a near-neutral intracellular pH. Exposure to external solutions with varying pH triggers extensive homeostatic responses, which in turn modulate cellular resistance. Figure 2b presents the results of treating onion membranes with solutions of different pH levels (pH = 4, 7, and 9). Under acid and base conditions, the Nyquist plots exhibit smaller phase angles at equivalent impedance magnitudes, while the system appears more stable under neutral stimulation. Because the measurements reflect the overall response of the inner epidermal cell, changes and movements in both the cell membrane and cell wall contribute to the observed phase shifts. Further experimental studies are required to explain the specific mechanisms involved.

### 3.3. Ion Liquid

Figure 2c shows the experimental results of PEIS measurements performed on onion inner membranes treated with KCl solutions at different concentrations. The KCl concentrations tested were 0.1 mol/L, 0.2 mol/L, 1/3 mol/L, and 1 mol/L, representing ionic stimuli ranging from below to above cytoplasmic concentrations.

At high frequencies, pronounced noise in the current response reflects cellular instability under high-frequency stimulation. Under 0.1 mol/L KCl, the impedance exhibited a relatively large phase angle, which progressively decreased as the KCl concentration increased.

A portion of the impedance of onion inner epidermal cells is contributed by vacuoles. The size of the vacuoles has a measurable influence on resistance, while distances among the vacuolar membrane, cell membrane, and cell wall affect capacitance values. The coupling of these two factors with other impedance elements may establish a mapping relationship between the equivalent circuit and vacuole dimensions. When external osmotic pressure exceeds that of the vacuole, onion cells undergo water loss, resulting in plasmolysis and a marked reduction in vacuolar volume. By adjusting the KCl concentration in the external medium, the osmotic pressure can be modulated, thereby altering vacuole volume and enabling fine-tuning of cellular impedance. This mechanism effectively transforms onion inner epidermal cells into precisely tunable impedance elements, facilitating their integration into circuits for implementing specific reconfigurable functions.

### 3.4. Equation

In current studies, equivalent circuits are commonly used to analyze the impedance of plant cells, providing a useful representation of their electrical characteristics to a certain extent [24,25]. Most research on equivalent circuits in plant cells has focused on analyzing individual electrical components or elements, emphasizing the equivalent resistance or capacitance effects of structures such as cell fluid and the cell wall [10,26]. We first observe that analogous impedance definitions exist across different physical domains, such as electrical impedance and mechanical impedance.

In the electrical field, impedance can be defined as

(1) $$Z_{e}=\frac{V}{I}$$

which is the ratio of voltage to current. Through the standard transformation in electrical physics, this definition can be equivalently expressed in terms of real and imaginary components.

(2) $$Z_{e}=R+jX$$

where the *R* represents the energy dissipation part and the *X* represents the energy storage part. Equation (2) is usually applicable in linear time-invariant systems, so it has limitations in describing living biological systems.

In fluid mechanics, the definition of flow impedance is

(3) $$Z_{f}=\frac{∆P}{Q}$$

where ∆P is the pressure difference and *Q* is the flow rate. This definition is similar to the electrical impedance in Equation (1). This can be understood as the ratio of a generalized force to a generalized flow. Similarly, the definition of mechanical impedance in classical mechanics follows the same trend

(4) $$Z_{m}=\frac{F_{0}}{v_{0}}e^{-ja}$$

which represents the ratio of force to velocity. This definition is also the ratio of a generalized force to a generalized flow, which is the theoretical basis for integrating the three physical fields into a unified impedance framework.

Given the inherent complexity of biological systems, multiple such systems are inevitably intertwined. Traditional impedance formulations primarily focus on electrical systems, typically either converting contributions from other domains into equivalent electrical terms or neglecting them altogether. The complexity of plant cell structure is often overlooked. In contrast, our hybrid equation directly considers the distinct contributions of different domains that collectively determine the overall impedance of biological systems. Under this conceptual framework, we generalize and unify the definition of impedance to encompass multiple physical domains, aligning more closely with the modeling trends in semiconductor synthetic biology. Plant resistance is determined by multiple factors, including cell structure, intracellular chemical reactions, intracellular fluid flow, and cell expansion. Based on an analysis of the structure of simple plant cells, we define impedance Z as a generalized impedance considering electrical, mechanical and fluid mechanical fields. We integrate Equations (2)–(4) into a novel impedance equation for plant cells [23].

(5) $$Z=K_{1}\left(R+Xi\right)+K_{2}\frac{∆P}{Q}+K_{3}\frac{F_{0}}{v_{0}}e^{-ja}$$

where

$K_{1}(R+Xi)$ is the electrical impedance component of the cells. $K_{1}$ is a constant related to the electrical properties, and R+Xi represents the impedance of the plant’s electrical equivalent circuit.

$K_{2}\frac{\Delta P}{Q}$ is the fluid impedance component, which is influenced by fluid flow such as that of cell fluid within the plant. $K_{2}$ is a constant related to fluid impedance.

$K_{3}\frac{F_{0}}{X_{0}}e^{-ia}$ is the mechanical impedance component, affected by cell movement, expansion, and contraction. $K_{3}$ is a constant related to mechanical impedance.

The physical meanings of the constants, such as $K_{1}{,K}_{2},\mathrm{and}K_{3},$ represent the relative ability of these three impedance components to modulate the overall impedance. For instance, the larger the mechanical impedance constant ($K_{3}$) is, the greater the overall impedance change will be due to a small mechanical variation.

According to the real and imaginary forms of impedance, the equation can be transformed into

(6) $$\left(K_{1}R+K_{2}\frac{∆P}{Q}+K_{3}\frac{F_{0}}{v_{0}}\cos\left(-a\right)\right)+\left(K_{1}X+K_{3}\frac{F_{0}}{v_{0}}\sin\left(-a\right)\right)i$$

which is helpful to further analyze various properties of the impedance in the complex plane.

Conventional equivalent circuit models are confined to fitting experimental results using electrical components. Due to the complexity of cells, such equivalence often suffers from ambiguous physical interpretability or yields limited accuracy. Many biochemical processes within cells cannot be adequately represented by simple electrical elements. To address this limitation, we introduce mechanical and fluid mechanical contributions into our framework, aiming to interpret impedance from a physically grounded perspective.

Our equation relies primarily on inference from existing experimental data and integration with established theoretical frameworks. The precise extraction of generalized impedance is still difficult using current techniques and concepts, so we do not fit this model to the experimental electrical impedance data. Microscopic observations and solution treatments confirm that mechanical components and fluid mechanical components constitute some factors contributing to the overall impedance. Precise quantitative verification may still need to be gradually carried out with the further development of this field.

### 3.5. Electrical Modulation Characteristics

The modulation of electrical properties constitutes an essential part of constructing computing systems. Currently, research on computing systems using biological materials remains at the preliminary stage of fundamental component assembly, where a thorough understanding of electrical modulation of cells is critical for accurately modeling these basic building blocks.

The impedance measurements reveal that plant cell impedance can change with both the frequency of the applied signal and the external ionic environment. This frequency-dependent behavior implies that when an electrical current containing multiple frequency components passes through plant cells, its spectral composition is altered. Motivated by this observation, we conduct electrical modulation experiments to verify and characterize the frequency-selective properties of plant cells, thereby inferring their potential capacity for signal processing.

Nevertheless, the realization of output conditioning by plant cells ultimately depends on the subsequent exploration and rational design of biomaterials beyond the scope of the present work. Direct experiments that change frequency components are conducive to the further development of this field.

#### 3.5.1. Experimental Setup

For complex signal analysis, we treated onion inner epidermal cells as circuit components and investigated their characteristics and electrical modulation capabilities. A segment of a human voice signal was recorded, and its waveform was converted into an electrical signal to serve as the input to a 20 × 20 mm onion cell sample. Unlike a fixed-frequency sine wave, a voice signal contains multiple frequency components and exhibits a complex spectral structure. The use of complex signals increases the complexity and randomness of the input, providing a more effective means to reveal the modulating ability of onion cells and to resemble real-world applications.

We recorded a human voice, processed it into a suitable electrical signal using MATLAB 2022a, and output it via a waveform generator to serve as the input for the cell system. The PCB was employed for signal transmission, and the onion cell membrane was placed on it. The step of the onion membrane operation was the same as the EIS impedance experiment. Through the PCB, two opposing electrodes were connected to the input, while the other two were connected to the output. The output was recorded using an oscilloscope.

To investigate the influence of external stimuli, the onion inner epidermal cells were treated with different concentrations of KCl, and the resulting outputs were recorded. We also treated the cells with solutions of different pH levels.

Considering the intracellular potassium ion concentration of onion inner epidermal cells, the equivalent concentration corresponding to the overall cellular osmotic pressure was approximately 100–200 mmol/L. According to osmotic experiments on onion inner epidermal cells, the limit of cell survival in an ion solution treatment reaches about 4 mol/L [27]. In our experiments, the KCl concentrations used were within the range that ensures cell viability. A KCl concentration of 1 mol/L is considered high for these cells, inducing obvious plasmolysis and morphological changes, while the other tested concentrations fall within the viable range.

To further quantify the input–output relationship, transfer function and coherence analyses were conducted as described below.

Transfer function analysis was performed in R using a Welch-averaged periodogram method to reduce the influence of random noise [28]. Input and output time series were aligned by common timestamps and preprocessed by linear detrending and mean removal. A Hann window was applied to each segment prior to spectral estimation. The transfer function was estimated using the $H_{1}$ estimator [29]:

(7) $$H_{1}\left(f\right)=\frac{S_{yx}(f)}{S_{xx}(f)}$$

where $S_{yx}\left(f\right)$ is the averaged cross-spectrum between the output and input signals and $S_{xx}(f)$ is the averaged autospectrum of the input. Transfer gain was defined as ${|H}_{1}\left(f\right)|$. The amplitude–frequency characteristic was expressed on the decibel (dB) scale as $20{log}_{10}|H(f)|$, allowing attenuation and amplification to be represented on a logarithmic scale.

Magnitude-squared coherence was computed as [30]:

(8) $$\gamma^{2}\left(f\right)=\frac{{|S}_{yx}\left(f\right){|}^{2}}{S_{xx}(f)S_{yy}(f)}$$

where $S_{yy}\left(f\right)$ is the averaged autospectrum of the output. Spectral estimation used a segment length of 100,000 samples with 75% overlap, yielding a frequency resolution of 50 Hz/bin. For visualization only, spectra were lightly smoothed using a centered moving average with a window width of 7 points.

#### 3.5.2. Results

**Figure 3:**
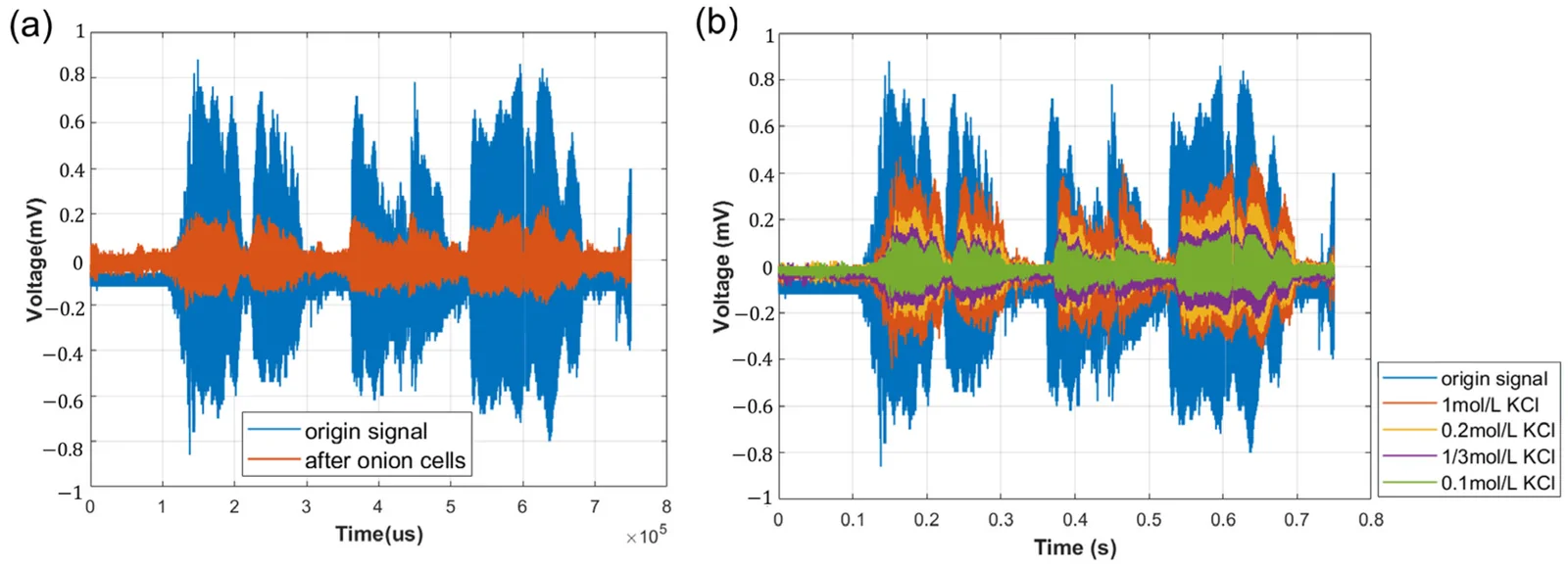
(**a**) Input and output time-domain waveforms of onion inner epidermal cells without KCl stimulation; (**b**) input and output time-domain waveforms under varying KCl concentrations.

After the waveform was applied to the onion cells, the output exhibited significant changes in both amplitude and frequency-domain components. Figure 3a shows the input and output time-domain waveforms without KCl stimulation, where clear attenuation of the signal amplitude can be observed.

Figure 3b further illustrates the corresponding waveforms under varying KCl concentrations, demonstrating that external ionic conditions significantly influence the signal response. The amplitude was notably attenuated, consistent with the presence of resistive components within the cellular impedance.

To quantitatively evaluate the measurement reliability of the PCOC platform, we utilized the approximately 100 ms silence segment before the onset of the voice input as an estimate of the system noise. By comparing the power spectral density between this silence segment and the effective signal segment, we estimated the system signal-to-noise ratio (SNR) under different ionic conditions. Across the five conditions with different KCl concentrations, the mean SNR was 18.9±4.2 dB. In the 1000–2000 Hz frequency range, the SNR reached 42.5 dB under 1 M KCl treatment. These results demonstrate that the platform is sufficient to resolve the impedance modulation responses by onion inner epidermal cells.

**Figure 4:**
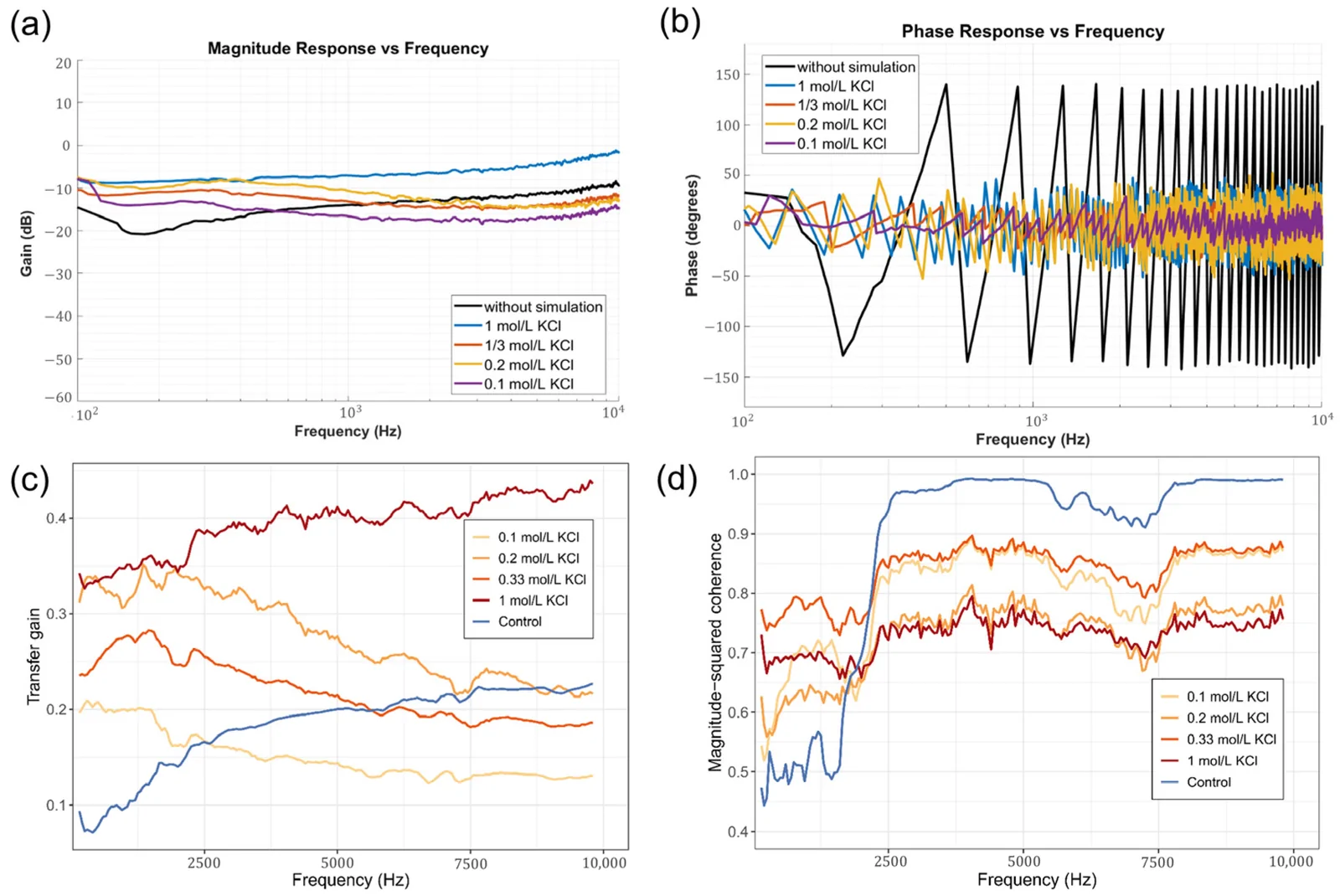
(**a**) Frequency-domain gain response under varying KCl concentrations; (**b**) phase-frequency response under varying KCl concentrations; (**c**) transfer gain under varying KCl concentrations; (**d**) magnitude-squared coherence under varying KCl concentrations.

The frequency response behavior indicates that the gain of onion inner epidermal cells varies with frequency, exhibiting distinct selectivity for different frequencies. Exposure to different external ionic stimuli produces unique characteristic curves in the onion epidermal layers. As shown in Figure 4a–c, the transfer gain and frequency-domain response exhibit distinct variations under different KCl concentrations, indicating frequency-selective behavior.

High concentrations of KCl (1 mol/L) induced pronounced plasmolysis in the onion inner epidermis, reducing vacuole dimensions and increasing the distance between vacuolar membranes of adjacent cells. These changes had a measurable impact on capacitive and RC circuit components and resulted in a significant modification of the transfer function. The magnitude response curve indicates that the conductivity of onion cells increases under 1 mol/L KCl stimulation.

In contrast to high-concentration conditions, more moderate ionic environments produce different response patterns. As shown in Figure 4a, stimulation with KCl solutions within the viable range (0.1, 0.2, and 1/3 mol/L) produced gain curves that were similar in overall shape, with differences primarily reflected in conductivity as a function of concentration. The selectivity for specific frequencies was also altered compared to the unstimulated condition.

The transfer gain shown in Figure 4c exhibits a clear dependence on KCl concentration compared to the unstimulated condition. The 1 mol/L KCl condition shows the highest transfer gain across the measured frequency range. Notably, the dependence on KCl concentration is not monotonic. The gain at 0.33 mol/L is lower than that at 0.2 mol/L in most cases, suggesting complex, nonlinear modulation of signal transmission by ionic conditions.

The coherence analysis in Figure 4b confirms the reliability of the measured signals. The coherence analysis further characterizes the relationship between input and output signals. In the control condition, coherence remains high above 2500 Hz (approximately 0.97–1.0), indicating a highly linear and predictable input–output relationship in this frequency range. The sharp increase near 2000 Hz likely corresponds to the lower boundary of the dominant energy band of the voice signal. Under all KCl-treated conditions, coherence plateaus at lower values (approximately 0.75–0.88 above 2500 Hz), significantly below the control. This reduction indicates that KCl stimulation introduces nonlinearity and variability into the cellular response, decreasing the predictability of the output from the input. Below 2 kHz, coherence remains low across all conditions, suggesting weak or less consistent signal transmission in this frequency range.

In addition, the phase-frequency response in Figure 4d reveals complex phase variations, although strong oscillations make quantitative interpretation challenging.

**Figure 5:**
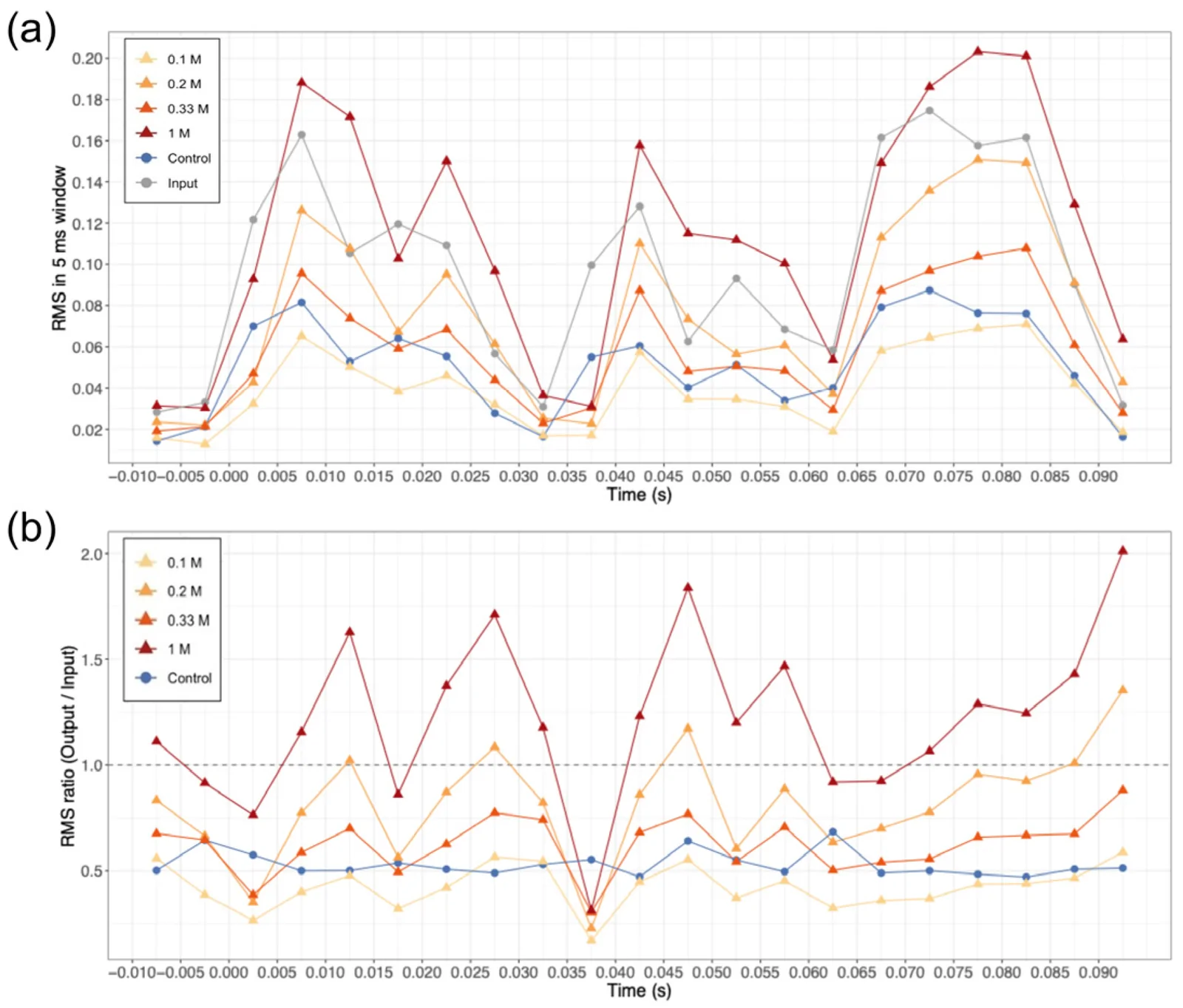
Graphs showing (**a**) 5 ms window RMS energy of input and output signals at varying KCl concentrations and (**b**) time evolution of the RMS ratio (output/input) under different KCl concentrations.

Time-domain root mean square (RMS) analysis further revealed nonlinear modulation of cellular energy transfer efficiency by KCl concentration. As shown in Figure 5a, the RMS energy of the output varies significantly with KCl concentration. Furthermore, Figure 5b presents the temporal evolution of the RMS ratio (output/input), highlighting nonlinear energy modulation and the dependence of energy transfer efficiency on ionic conditions. In the high-concentration group, the output RMS exceeded the input, whereas the low-concentration group exhibited consistent energy attenuation. Across all experimental groups, the output signal remained highly synchronized with the input signal, demonstrating the real-time responsiveness of the cellular system.

To quantitatively evaluate the nature of this modulation, both linear and nonlinear (quadratic) models were applied to fit the relationship between KCl concentration and output RMS energy. The quadratic model yielded a lower fitting error (0.00027131) compared to the linear model (0.00031956), indicating that the observed dependence cannot be fully captured by a linear approximation. This improvement in fitting accuracy supports the presence of nonlinear modulation mechanisms governing energy transfer efficiency under varying ionic conditions. This conclusion is also supported by the frequency-domain curves in Figure 4.

Changes in pH significantly affected cell viability. Under extreme pH conditions, cellular function was compromised, often leading to rapid cell death. Under such conditions, the output became severely attenuated, making effective signal transmission infeasible.

#### 3.5.3. Interpretation and Mechanisms

The observed attenuation of signal amplitude can be attributed to the resistive components of cellular impedance. Since the impedance of cells includes resistive elements, the reduction in amplitude is consistent with electrical energy dissipation during signal transmission.

The gains and selective response in Figure 4 indicate that onion epidermal cells behave as frequency-selective systems. The variation in the transfer function under different ionic conditions suggests that external stimuli can modulate the electrical properties of the cells.

The variation in transfer gain under different KCl concentrations can be attributed to changes in the effective electrical conductivity of the cellular system. High KCl concentrations increase the availability of mobile ions, thereby reducing resistive components within intercellular pathways and enhancing signal transmission efficiency. This is consistent with the higher transfer gain observed under 1 mol/L KCl conditions.

The dependence of transfer gain on KCl concentration is not monotonic. This suggests that multiple competing mechanisms are involved, including ion channel regulation, membrane polarization effects, and structural changes such as plasmolysis. While increased ionic strength enhances conductivity, excessive KCl may disrupt normal membrane function and alter capacitive coupling, leading to complex and nonlinear modulation of transfer characteristics.

The coherence results provide further insight into the linearity of the cellular response. In the unstimulated condition, the high coherence observed at higher frequencies indicates a predominantly linear and time-invariant relationship between input and output signals.

In contrast, the reduced coherence under KCl stimulation suggests the emergence of nonlinear and time-varying behavior within the cellular system. Such behavior may arise from dynamic biological processes, including stochastic ion channel gating, time-dependent membrane polarization, and intracellular ion redistribution. These processes introduce variability and reduce the predictability of the output signal based on the input.

The consistently low coherence observed at lower frequencies may reflect intrinsic limitations in low-frequency signal transmission, potentially due to capacitive effects of the membrane or slower ionic response dynamics.

The RMS behavior indicates that the system does not simply attenuate signals but redistributes energy over time. As a measure of energy integration and normalization over a defined time window, RMS reflects the temporal redistribution of energy, suggesting that onion cells modulate both the energy distribution and frequency composition of the input. Specifically, the energy of the output became more concentrated within particular time windows, accompanied by selective enhancement in high-frequency harmonic components.

**Figure 6:**
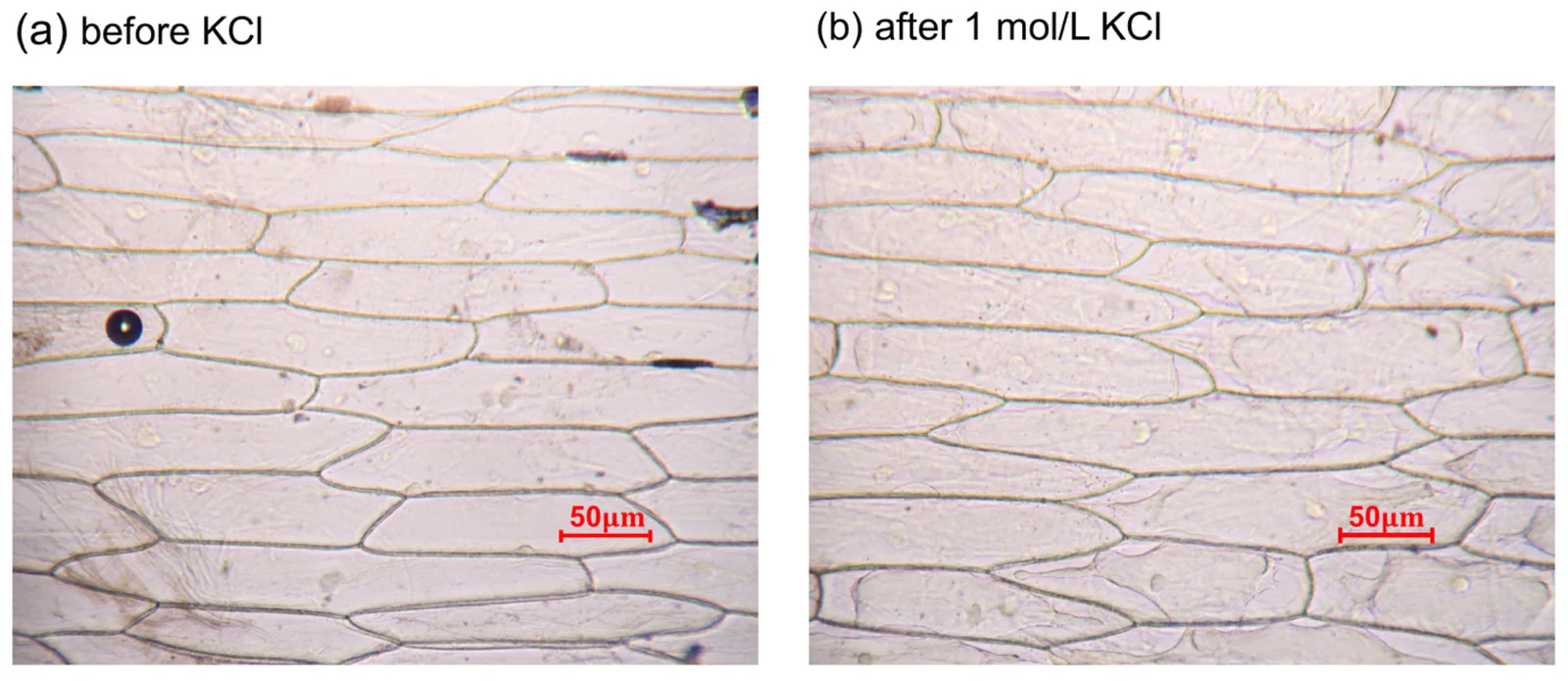
Morphology of onion cells.

Within the viable range, KCl enables tunable modulation of impedance and signal transmission properties. Morphological changes in the onion cells were observed under the microscope, as shown in Figure 6. In contrast, extreme conditions, such as high KCl or extreme pH, lead to structural damage and loss of functional signal processing capability.

The experimental results described above indicate that onion inner epidermal cells exhibit frequency-dependent signal responses under various external stimuli. Plant tissues possess intrinsically complex structures, and the detailed mechanisms remain unclear. The overall changes in electrical properties may be caused not only by physical effects but also by biological regulation. We interpret the impedance changes from the perspective of their impact on electrical signals and attempt to identify the possible mechanisms based on the obtained frequency response behaviors.

From a structural perspective, many cellular components can function as resistive elements. The equivalent circuit of the cell membrane includes distributed capacitance and resistance, forming RC networks that generate relaxation effects, alter transconductance, and adjust gain.

Based on this equivalent circuit framework, external stimuli are expected to modulate the electrical response of the system. KCl stimulation within the viable range induces changes in the intracellular environment and alters the state of ion channels in the cell membrane, potentially modifying the specific properties of the membrane. This leads to shifts in characteristic frequency, including changes in the peak of the transfer function and its amplitude. The nonlinear relationship between gain and KCl concentration may arise from different ion channel states induced by transmembrane ion concentration gradients. Similarly, other external chemical stimuli can also modulate the electrical properties of the cell membrane. Mild pH variations can also modulate impedance, although such effects may exhibit partial irreversibility depending on the extent of cellular damage.

When the KCl concentration exceeds the viable range, structural changes become dominant. At high concentrations, KCl induces plasmolysis, reducing vacuole size and increasing separation between cellular structures. According to the microscope image in Figure 6b, the average area of the inner epidermal cells of onions under the 1 mol/L KCl treatment is 6237.6 ${\mu m}^{2}$, with the average vacuole size 4278.1 ${\mu m}^{2}$. The average plasmolysis index reaches 68%. The cellular structural changes are sufficiently evident to modulate the impedance. This may create conductive pathways between the cell wall and membrane, alter capacitive coupling, and significantly modify impedance characteristics.

In addition to membrane-level effects, other structural components of plant cells also contribute to the observed electrical behavior. Plant cells also possess cell walls, where diffusion involving specific ions or interfacial polarization in intercellular spaces or the cell wall itself can further contribute to differences in the transfer function. Additional factors, including structural resonance, ion channel coupling, and mechanoelectrical interactions, further influence amplitude and frequency response.

Given that the internal structure of plant cells, including RC circuits, structural resonance, ion channel coupling, and mechanoelectrical reactions, all influence both amplitude and the transfer function, the underlying mechanisms governing transfer function variation remain difficult to describe precisely. These mechanisms collectively contribute to the observed modulation of impedance and the transfer function. Due to the complexity of intracellular regulatory processes and physical effects in cells, precise control and prediction of the transfer function remain challenging.

Despite these limitations, external environmental factors still provide a means to modulate cellular electrical performance. The variation enables the adjustment of impedance through external stimuli such as ionic solutions, highlighting the potential of using plant cells to construct tunable impedance-based electrical components.

## 4. Discussion

### 4.1. EIS Method

Electrical signals play an important role in both intracellular and intercellular communication in plant cells. To study the electrical responses of plants, various methods ranging from DC to AC have been applied to observe and explain plant reactions to electrical stimulation, often treating plants as components of circuits [10,31]. While these methods effectively explore plant responses to specific voltages, voltage-induced charge accumulation can affect stability and obscure certain electrical properties, a gap that electrochemical impedance spectroscopy (EIS) helps to fill [31,32].

Other methods have also been developed and applied to explore the electrical properties of cells, including bio-impedance measurement and microelectrode arrays (MEAs) [33,34]. Bio-impedance measurement directly introduces a wire to measure potential and obtain impedance and was commonly used in early explorations [33]. Early implementations using constant current sources could lead to voltage accumulation, potentially damaging cells and affecting measurement outcomes. The MEA method uses electrode microarrays to record cell field potentials and offers the advantage of being non-invasive. Cells cultured directly on MEAs enable simultaneous observation of electrophysiological activity and morphology. This technique is particularly well suited for nerve cells [34,35], cardiomyocytes [36], retinal cells, and similar cell types. The information obtained via MEAs is primarily limited to field potentials. Compared to MEAs, electrochemical impedance spectroscopy (EIS) is another non-invasive method that obtains dynamic electrical data through small-amplitude perturbation current analysis. This approach offers greater advantages for analyzing intracellular reactions.

EIS has been widely used to investigate reaction processes, particularly in electrochemical contexts such as electrocatalysis, as well as to characterize conductive materials like porous electrodes and coatings. Beyond direct interactions between cellular components and electronic transducers, impedance variations can also be used to monitor intracellular activities and stimulus-induced responses [31,37].

In this work, our design integrates the precise fluid control of microfluidics, the electrical signal controllability of chip-based systems, and the ease of handling of plant cells. This combination offers broad application potential for studying plant cell impedance and exploring electrical characteristics. The multilayer, reconfigurable design allows the chip to be adapted to different experimental conditions and stimulation types. Additionally, the integration of a PCB enables accurate and convenient application of EIS signals and reliable acquisition of feedback data.

To ensure close contact between plant cells and the PCB, we used a flat PDMS layer, which introduced limitations in controlling stimulus liquids and led to instability under pressure changes. The design of microfluidic devices for EIS measurements requires further optimization to accommodate varying experimental conditions. Since our focus is on impedance analysis of plant cells, direct contact between the ion solution and the electrodes creates parasitic conductivity, which significantly compromises measurement accuracy. This issue complicates the design of the liquid control layer. To minimize direct ion conductivity, we adopted a flat PDMS layer to reduce liquid retention within the onion cell layer. Further improvements to the liquid control layer of the microfluidic chip are needed to enable flexible stimulation and enhance the adaptability of the chip. A more advanced PDMS design, using patterned structures instead of a flat contact, could help suppress direct ionic conductivity, allow flexible control of solution-based stimulation, and maintain cell viability.

The stripped inner epidermal cells of onions gradually lose viability over time, which affects EIS measurements and imposes stringent requirements on experimental timing and technique. Different electrode geometries can also lead to variations in electrical field distribution, suggesting that electrode design can be optimized and systematically tested.

Plant cells harbor a wealth of unexplored electrical properties and information. As chip-based technologies continue to improve, more plant cell types are expected to be introduced as experimental materials. The combination of EIS with multi-part and multilayer chips holds promise as a powerful tool for further exploration. In future work, integrating this chip with microfluidic channel designs could enable the construction of flexible, controllable devices suitable for cell detection under a variety of liquid conditions, thereby improving the efficiency of plant cell-on-chip (PCOC) platforms.

### 4.2. Electrical Modulation of Plant Cells

The equivalent circuits of plant cells are impacted by a range of complex factors, including cellular structure, internal chemical reactions, and ion exchange. These circuits cannot be adequately described by a simple RC circuit or a single model. The gain curve results show differences across various experimental conditions within the 100 to 10,000 Hz range, with an upward trend observed at higher frequencies.

In the absence of stimulation and within a specific range of KCl solution treatment, a downward trend is observed at lower frequencies, with the unstimulated condition showing a distinct local minimum. We hypothesize that a component of the plant cell equivalent circuit is frequency-dependent and modulated by the state of ion channels or the transmembrane ion concentration gradient. This component may become dominant or non-negligible under certain conditions, leading to changes in the gain curve. From a structural perspective, the diffusion delay of ions across the membrane manifests as Warburg impedance at high frequencies. Its phase-lead characteristic, analogous to an inductive element, counteracts the capacitive attenuation of the membrane capacitance, resulting in a frequency-dependent gain increase. Parasitic inductance from the electrode or layered structure of our device may also influence the above interpretation. Further experiments and device refinements are needed to explore the true equivalent circuit of plant cells. In subsequent studies, the continued development of experimental methods and equipment may reveal additional factors and enable new analytical approaches.

Our experiments demonstrate that the inner epidermal cell membrane of onions exhibits selectivity for electrical signals at different frequencies and can modulate its impedance in response to external environmental factors such as KCl concentration and pH. The tunable impedance characteristic suggests that under precisely controlled conditions, plant cells can serve as a component of bioelectrical devices, potentially participating in signal processing or computing tasks within hybrid bioelectronic systems.

### 4.3. Prospects of Plants as Electrical Components

Our work explored the feasibility of modulating the impedance and frequency characteristics of plant cells by varying KCl concentrations, demonstrating that plant cells can function as electrical components that can be programmed by the ionic environment. The experiments clearly revealed the nonlinear nature of the cellular impedance response and electrical modulation rules. Through rational design of the PCOC platform, environmental variables such as external ionic concentration can be incorporated as independent control modulators into the computing architecture, enabling modulation of the electrical properties of cells. In this sense, plant cells can be analogized with rheostats, whose impedance can be actively adjusted via external control. But the realization of this concept in practical biohybrid computing systems will ultimately depend on further design and development.

Plant cells are the core of the PCOC platform. Current studies using PCOC usually aim to detect plant growth by exploring more mechanisms between various physiological traits [38,39,40]. But based on the synthetic bioelectrical circuitry concept, what humans fabricate and design will transform from sensors for plant detection to plants for signal processing. Biological materials will become the components of hybrid integrated bioelectrical chips.

Decellularized plants, in which cellular activity is removed while the natural scaffold is preserved, have been extensively employed as chip frameworks for engineering biological models. For example, a decellularized plant-derived vasculature-on-a-chip enables the study of interactions with breast cancer spheroids [41]. Similarly, a decellularized plant-based air–liquid-interface-on-a-chip has been developed to model respiratory responses to particulate matter aerosol exposure [42]. These examples underscore the utility of plants as functional chip components, even in the absence of living cells. The dynamic responsiveness of living plant cells remains unexploited in these decellularized platforms, yet it holds untapped potential for active sensing and information delivery.

These advances demonstrate the remarkable versatility of biological materials in their potential applications. The design of bioelectronic components from such materials involves diverse development strategies depending on the intended functionality. Whether employed as structural substrates or functional active elements, plant cells hold substantial value for long-term participation in the evolution of biohybrid computing systems.

This hybrid integrated bioelectrical concept falls within the field of semiconductor synthetic biology, aiming to harness biological advantages to help achieve computing functions. SemiSynBio is still preliminary, and the study of impedance in plant cells falls within the stage of exploring fundamental building blocks for biohybrid computing systems. By outlining the potential future directions, we hope to inspire further research and accelerate progress in this emerging field.

The frequency-dependent responses exhibited by plant cells suggest the presence of multiple effects and processes within them. If targeted enhancement or suppression of specific frequency responses can be achieved through methods such as genetic engineering, chemical modification, or physical constraints, it may become possible to construct cell-based signal processing modules with functions. Such modules can be integrated with traditional microelectronic circuits to form bio-semiconductor hybrid systems, offering a potential pathway toward novel computing architectures that are low-power and environmentally responsive.

Additionally, compared with animal cells, plant cells offer advantages such as stable cell walls, ease of batch acquisition, and no requirement for strict aseptic handling, making them more suitable for device integration in open or semi-open environments. In future work, the specific impedance responses of different plant cell types can be further explored. By integrating microfluidics and planar electrode array technologies, a high-throughput, reconfigurable cell membrane device platform could be realized, advancing semiconductor synthetic biology in the directions of signal processing and sensory computing.

Expanding from plant cell models to animal cell systems is a feasible next step in the exploration of cells as active electronic components, offering the potential for more dynamic and complex functionality in synthetic bioelectronics [43,44,45]. These experiments in plants, animal cells and other biological materials are necessary steps toward SemiSynBio development [46,47,48].

Despite its promising prospects, this field still faces core challenges, including maintaining long-term biocompatibility, developing suitable computational frameworks, and ensuring system stability and repeatability. Future research will require closer collaboration between microelectronics engineering and synthetic biology to advance the integration and application of cells as true, high-performance “living components” in next-generation intelligent systems and computing paradigms.

### 4.4. Improvement in the PCOC Platform

As an experimental platform, PCOC provides us with a methodology and a testing platform. In future studies, noise characterization and detection limit evaluation will be performed on the PCOC platform to further improve its reliability and resolution for cellular electrical characterization.

In future studies, more quantitative analyses of plasmolysis, cell viability, and membrane integrity may provide new insight into the mechanism of plant cell modulation characteristics since impedance is highly sensitive to cellular structures, metabolic states, and ion exchange rates. Current studies on the electrical properties of plant cells still lack precise performance analysis, including assessments of variability and the extent to which these properties are affected by cell viability. If such performance consideration is incorporated into the evaluation criteria for bioelectronic materials, the fundamental component layer of SemiSynBio would be further refined and advanced.

## 5. Conclusions

In this study, we designed and established a plant cell-on-chip platform integrating EIS to investigate the electrical properties of onion inner epidermal cells. The results demonstrate that cellular impedance can be modulated by cell dimensions and external stimuli, including ionic concentration and pH, causing distinct frequency-dependent responses. We derived an impedance equation for the hybrid system and analyzed both impedance and transfer function characteristics of onion cells under various stimuli, revealing tunable electrical properties.

These findings indicate that plant cells possess tunable electrical properties, positioning them as potential components in biohybrid circuits. The PCOC platform provides a controllable approach for detecting cellular responses in different ion environments, offering new insights into the electrical properties of plant systems. The tunability of impedance via external stimulation suggests a mechanism for integrating biological variability into circuit design.

Future work will focus on improving the device and extending the platform to other plant and animal cell models to explore broader bioelectronic applications. Our approach contributes to the emerging field of semiconductor synthetic biology (SemiSynBio), where living cells serve as functional elements in hybrid electronic systems. The development of hybrid electronic systems still needs more experiments, design, and exploration of mechanisms.

## Data Availability

The original contributions presented in this study are included in the Supplementary Materials. Further inquiries can be directed to the corresponding author.

## Supplementary Materials

The following supporting information can be downloaded at: https://www.mdpi.com/article/10.3390/mi17091015/s1.
